# Snack frequency, size, and energy density are associated with diet quality among US adolescents

**DOI:** 10.1017/S1368980023001635

**Published:** 2023-11

**Authors:** Gina L Tripicchio, Regan L Bailey, Adam Davey, Christina M Croce, Jennifer Orlet Fisher

**Affiliations:** 1 Center for Obesity Research and Education, Temple University, Philadelphia, PA, USA; 2 Institute for Advancing Health through Agriculture, Department of Nutrition, Texas A&M University, College Station, TX, USA; 3 Behavioral Health and Nutrition, University of Delaware, Newark, DE, USA

**Keywords:** Diet, Snacking, Adolescents, Behavioural health

## Abstract

**Objective:**

To evaluate snacking and diet quality among US adolescents.

**Design:**

Cross-sectional analysis examined snack frequency (snacks/day), size (kcal/snack) and energy density (kcal/g/snack) as predictors of diet quality using the mean of two 24-h dietary recalls. Diet quality was assessed using the Healthy Eating Index (HEI-2015, 0–100), a mean adequacy ratio (MAR, 0–100) for under-consumed nutrients (potassium, fibre, Ca, vitamin D) and mean percentage of recommended limits for over-consumed nutrients (added sugar, saturated fat, Na). Linear regression models examined total snacks, food only snacks and beverage only snacks, as predictors of diet quality adjusting for demographic characteristics and estimated energy reporting accuracy.

**Setting:**

2007–2018 National Health and Nutrition Examination Survey.

**Participants:**

Adolescents 12–19 years (*n* 4985).

**Results:**

Snack frequency was associated with higher HEI-2015 (*β* = 0·7 (0·3), *P* < 0·05) but also with higher intake of over-consumed nutrients (*β* = 3·0 (0·8), *P* ≤ 0·001). Snack size was associated with lower HEI (*β* = –0·005 (0·001), *P* ≤ 0·001) and MAR (*β* = –0·005 (0·002), *P* < 0·05) and higher intake of over-consumed nutrients (*β* = 0·03 (0·005), *P* ≤ 0·001). Associations differed for food only and beverage only snacks. Food only snack frequency was associated with higher HEI-2015 (*β* = 1·7 (0·03), *P* ≤ 0·001), while food only snack size (*β* = –0·006 (0·0009), *P* ≤ 0·001) and food only snack energy density (*β* = –1·1 (0·2), *P* ≤ 0·001) were associated with lower HEI-2015. Conversely, beverage only snack frequency (*β* = 4·4 (2·1) *P* < 0·05) and beverage only snack size (*β* = 0·03 (0·01), *P* ≤ 0·001) were associated with higher intake of over-consumed nutrients.

**Conclusions:**

Smaller, frequent, less energy-dense food only snacks are associated with higher diet quality in adolescents; beverages consumed as snacks are associated with greater intake of over-consumed nutrients.

Paediatric obesity is a pressing public health issue, and poor diet quality is a key modifiable risk factor^(1–3)^. Dietary patterns in adolescence track into adulthood and can impact long-term dietary behaviours and chronic disease risk, underscoring the significance for prevention^(4)^. US adolescents have the lowest overall diet quality of any age group, and the emergence of poor eating behaviours like meal skipping is typical during this developmental period^(5–7)^. Dietary intake among US adolescents is also characterised by low intakes of a constellation of under-consumed nutrients as well as excessive intakes of over-consumed nutrients, particularly added sugar^(8)^. One eating behaviour that remains understudied as a key driver of suboptimal intake in this age group is snacking.

Snacking (i.e. eating in between meals) is ubiquitous among US adolescents; in 2018, snacking was reported by approximately 89 % of adolescents and provided approximately 22 % of daily energy^(9–11)^. Current dietary guidelines recommend healthy snacking choices for children in order to meet their nutrient needs^(12)^. However, snacking remains a controversial topic and evidence to date has been equivocal on how snacking impacts dietary exposures among adolescents. Some studies among adolescents have shown that snacking is associated with higher daily energy, lower intakes of fruits and vegetables, higher intakes of sugary drinks and fast foods, and poorer overall diet quality^(13,14)^. However, other studies have shown that snacking can contribute positively to overall diet, but associations vary based on participant characteristics (e.g. gender) and how snacking is defined^(15,16)^. Snacking is commonly defined using frequency (i.e. number of snacks per day), but other metrics have included time of day (e.g. food eaten outside of mealtimes), percentage of total energy (e.g. < 15 % total energy) and types of foods (e.g. cookies, chips)^(13–15,17–19)^. For example, work by Murakami *et al*. examined the associations between eating frequency and diet quality in US children and adolescents using three definitions for snacking: (1) intake occasions < 15 % of total energy, (2) self-defined eating occasions and (3) occasions based on time^(15)^. When snacks were defined as a percentage of total energy, snack frequency was positively associated with diet quality. However, snacking based on self-defined occasions or time showed no association with diet quality. In another study, snack energy density was examined in British adolescents using similar definitions and found that snack energy density was inversely associated with overall diet quality^(20)^. Given this preliminary work and methodological limitations in the current literature, there is a need to examine the associations of snacking and diet quality using comprehensive definitions that include frequency, size, and energy density and models that control for other snacking parameters^(15,19,21)^. This approach has been applied in previous work among preschool children and found that snack frequency was associated with higher diet quality while snack size and energy density were associated with lower diet quality^(22)^. Similarly, snack frequency, size, and energy density were examined in relation to weight status among adolescents and results found that more frequent, larger, and more energy-dense snacks were associated with higher weight status in this age group^(23)^. However, to date, these snacking parameters have not been examined in relation to diet quality in US adolescents. Addressing this gap has potential public health significance since diet quality is lowest among US adolescents, and evidenced-based policy recommendations and dietary guidelines around snacking for this life stage are lacking.

Thus, the research presented herein addresses current gaps in the literature by comprehensively evaluating multiple parameters of snacking and diet quality among US adolescents over the most recent decade. The objective for this research is to examine three distinct snack parameters: (1) snack frequency (snacks/day); (2) snack size (kcal/snack) and (3) snack energy density (kcal/g/snack) and the association with three dimensions of diet quality: (1) overall diet quality assessed using the 2015 Healthy Eating Index (HEI-2015); (2) the mean adequacy ratio (MAR) of intake of four under-consumed dietary components of public health concern: potassium, fibre, Ca, and vitamin D and (3) the mean intake of over-consumed nutrients as a percentage of recommended limits: added sugar, saturated fat, and Na^(24)^.

## Methods

### Design and participants

Cross-sectional data from 4985 adolescents, aged 12–19 years, who participated in the 2007–2018 National Health and Nutrition Examination Survey (NHANES) were included in this study. NHANES is an ongoing nationally representative study of the nutrition and health status of the civilian, non-institutionalised US population. NHANES uses complex, multistage probability sampling to identify clusters of household and individual participants from counties (primary sampling unit). Data are collected by trained study staff. During an in-person visit, participants complete a medical history, an interview, a physical examination, and the first of two 24-h dietary recalls. A second 24-h dietary recall is collected via telephone. NHANES participants provide informed consent and the National Center for Health Statistics Research Ethics Review Board approves the study protocols. Additional details about NHANES study design and data collection procedures are detailed elsewhere^(25)^. Only adolescents with 2 d of dietary data were included in these analyses (initial sample *n* 6117; see online supplementary material, Supplemental Figure 1). Participants were excluded if they were missing height/weight data (*n* 79), had a diagnosis of diabetes (*n* 44), reported use of medication known to impact hunger, appetite or weight status (*n* 93), did not report any snacking (*n* 435) and/or were missing covariate data (*n* 295 missing head of household (HH) marital status; *n* 186 missing HH education status), yielding a final analytic sample of 4985.

### Dietary intake

Dietary data were collected via two 24-h dietary recalls using the United States Department of Agriculture automated multiple-pass method^(26–28)^. Adolescents self-reported dietary intake. Eating occasions were characterised by participants using a pre-determined list of occasions (e.g. ‘breakfast’, ‘snack’ or their Spanish equivalents). An occasion-based definition of snacking was employed including all occasions of eating and drinking in between meals; snack occasions included those identified by participants as ‘snacks’ as well as those identified as ‘beverages’ or ‘extended consumption’^(6)^. Snack foods/beverages consumed at the same time were aggregated into a single eating occasion. The mean of two 24-h dietary recalls was used to calculate mean snacking frequency (number of snacks/day), mean snack size (kcal/snack occasion) and mean snack energy density (kcal/g/snack occasion). The Food Patterns Equivalents Database was used to convert the What We Eat In America dietary data using the corresponding data files for each cycle^(29)^. Dietary weights were used to adjust for dietary non-response, day of the week, and multiple NHANES cycles^(28)^. Consistent with our previous work, initial models were evaluated with and without snacks providing trivial energy (eating occasions < 5 kcal)^(22,23,30)^. Results were similar; therefore, trivial snacks occasions were excluded. Also, given the variation in energy density scores between foods and beverages (e.g. beverages have lower energy density than foods due to water content) and potential variation in contributions to diet quality, total snack categories were stratified to examine beverage only snacks and food only snacks.

### Dietary reporting accuracy

Following the methods in Murakami and Livingstone, the ratio of reported energy intake to estimated energy requirements (EI: EER) was used to assess dietary reporting accuracy^(31)^. EER were calculated using dietary reference intake equations based on age, gender, weight and physical activity level^(32)^. A ‘low active’ level of physical activity (≥ 1·4 to < 1·6) was assumed for all adolescents since objective physical activity data were not available and other surveillance studies among adolescents demonstrate insufficient/low physical activity trends in this group^(33,34)^. The EI: EER ratio was used as a covariate in the analyses to account for reporting error (rather than removing implausible cases), without biasing sample selection.

### Healthy Eating Index

The HEI-2015 was used to assess overall diet quality. HEI-2015 is an index used to assess the extent to which individuals are meeting their recommended nutrient intake, as specified by the Dietary Guidelines for Americans^(12,35)^. The HEI-2015 score consists of thirteen food group components that are scored based on ranges from 0 to 5 or 0 to 10 depending on the scale. Component scores are summed to create an overall score ranging from 0 to 100, with 100 representing complete alignment with the Dietary Guidelines for Americans, and a lower score representing poorer overall diet quality. The thirteen components are further categorised into adequacy components (i.e. dietary intake is encouraged) and moderation components (i.e. recommendations suggest limited consumption). Adequacy components include total fruits (includes 100 % fruit juice), whole fruits (includes all fruit except juice), total vegetables, greens and beans, whole grains, dairy, total protein foods, seafood and plant proteins, and fatty acids. Moderation components include refined grains, Na, added sugar, and saturated fats. For adequacy components, higher scores reflect higher intake. For moderation components, higher scores reflect lower intake. HEI was derived as a total continuous overall score for adolescents and subscale scores were also calculated.

### Mean adequacy ratio

A MAR was calculated to assess under-consumption of dietary components of public health concern^(36)^. Nutrient adequacy ratios were calculated for each nutrient based on the ratio of mean daily intake relative to the recommended intake (i.e. adequate intake for potassium; 14 g/1000 kcal for fibre and RDA for Ca and vitamin D)^(12,37)^. Values were truncated at 100, with 100 % indicating complete alignment with the recommendation. The MAR was calculated by averaging the nutrient adequacy ratios for all four nutrients. Higher MAR scores indicate greater daily intake of the four under-consumed nutrients, relative to the recommendations for those nutrients.

### Over-consumed nutrients

Intakes of added sugar, saturated fat, and Na were expressed as a percentage of recommended limits for each nutrient: recommended limits on added sugar and saturated fat are < 10 % daily energy based on the Dietary Guidelines for Americans and limits on Na are < 1800 mg if < 14 years and < 2300 mg if > 14 years, based on the tolerable upper intake level from the dietary reference intake^(12,37)^. The mean percentage of recommended limits was calculated for the three over-consumed nutrients and results were not truncated (i.e. percentages could be above or below 100 %); for instance, a score of 120 % would indicate that mean intake of added sugar, saturated fat, and Na was 120 % of the recommended limits.

### Demographics

Age (years), gender (male/female), and race and ethnicity (non-Hispanic White, Hispanic/Mexican American, non-Hispanic Black, other) were collected for the participating adolescents from the in-home interview. HH age, marital status, education level, and income were also collected. The ratio of income to poverty (PIR) was estimated using family income relative to the poverty threshold; families greater than 125 % of the poverty threshold were classified as high PIR and those less than 125 % of the poverty threshold were classified as low PIR^(38)^. Adolescent height and weight were objectively assessed by trained staff and used to calculate EER for energy misreporting (EI: EER) as noted above.

### Analysis

Survey weights were applied, and descriptive statistics were generated and presented as means (standard errors) for continuous variables and percentages for categorical variables. Multiple linear regression models evaluated the three snacking parameters (total snack frequency, size, and energy density) as predictors of each diet quality outcome (HEI-2015, MAR, over-consumed nutrients), adjusting for all other snacking parameters. Then secondary outcome models were stratified to examine food only snacks and beverage only snacks. Initial covariates were selected a priori based on prior work and existing literature and then backwards stepwise elimination was used to determine the inclusion of covariates (*P* > 0·1). Child age, gender, race, and ethnicity, HH education status, and HH marital status were retained, and all models were adjusted for survey cycle year and estimated energy reporting accuracy (EI: EER). Listwise deletion was used to remove cases for which key covariates were missing. Adolescents who had zero calories from snacks were also dropped from the models as snack size and snack energy density cannot be derived. All tests were two-sided, and significance was set at *P* < 0·05. All analyses were conducted in Stata (version 15.1; StataCorp).

## Results

Adolescents (*n* 4985) were 15·3 (0·05) years, 50·4 % female, 55·0 % non-Hispanic White, 22·8 % Hispanic or Mexican American, and 13·8 % non-Hispanic Black (Table 1). A majority of HH caregivers were 40 years or older (73·0 %), 71·8 % were married or partnered, 20·3 % had less than high school education, 51·7 % graduated high school or had some college, 28·0 % were college graduates, and 29·5 % had low PIR status. Most adolescents (92·6 %) consumed snacks, with only 7·4 % reporting no snack intake. On average, adolescents who snacked consumed 1·7 (0·02) total snacks per day (min 0·5 snacks, max 8 snacks), 282·3 (4·9) kcal per snack (min 5 kcal, max 2434 kcal) and a mean snack energy density of 2·6 (0·03) kcal/g/snack (min 0·1 kcal/g, max 6·3 kcal/g). Mean energy density of food only snacks was 3·4 (0·03) kcal/g/snack, and mean energy density of beverage only snacks was 0·6 (0·02) kcal/g/snack. In the stratified models, 4757 participants (95·4 %) consumed food only snacks and 3442 participants (69·0 %) consumed beverage only snacks.

**Table 1:** Demographic characteristics of US adolescents (*n* 4985), data from 2007–2018 National Health and Nutrition Examination Survey

	*n*	%
**Adolescents**		
Age, years		
M	15·3	
se	0·05	
Race		
Non-Hispanic White	1412	55·0
Mexican American/Hispanic	1693	22·8
Non-Hispanic Black	1201	13·8
Other	679	8·4
Gender		
Male	2505	49·6
Female	2480	50·4
EI: EER		
M	0·8	
se	0·007	
**Head of household**		
Age		
< 40 years	1524	27·0
40 years and older	3461	73·0
Ratio of income to poverty^*^		
Low	1759	29·5
High	2844	70·5
Education		
Less than high school	1350	20·3
High school graduate/some college	2565	51·7
College graduate	1070	28·0
Marital status		
Partnered	3379	71·8
Not partnered	1606	28·2

MEI:EER, ratio of energy intake to energy expenditure.

* Calculated by dividing family income by the Health and Human Services’ poverty guideline, specific to family size, as well as the year and state (*n* 4603).

### Associations of snacking with overall diet quality

Adolescents had a mean HEI-2015 of 45·2 (0·3) out of possible score of 100, indicating overall suboptimal diet quality (Table 2). After adjusting for the other snacking parameters as well as other covariates, total snack frequency was positively associated with HEI-2015 (*β* = 0·7 (0·3), *P* < 0·05) (Table 3), total snack size was inversely associated with HEI-2015 (*β* = –0·005 (0·001), *P* ≤ 0·001), and total snack energy density was not associated with HEI-2015 (*β* = –0·3 (0·2), *P* = 0·224). When examining food only snacks, frequency of food only snacks was positively associated with HEI-2015 (*β* = 1·7 (0·3), *P* ≤ 0·001). Food only snack size and food only snack energy density were inversely associated with HEI-2015 (snack size (*β* = –0·006 (0·0009), *P* ≤ 0·001; snack energy density *β* = –1·1 (0·2), *P* ≤ 0·001). When examining beverage only snacks, there was no association between beverage only snack frequency (*β* = –0·1 (0·6), *P* = 0·857), beverage only snack size (*β* = –0·002 (0·002), *P* = 0·342) or beverage only snack energy density (*β* = 1·1 (0·8), *P* = 0·151) and HEI-2015.

**Table 2:** Descriptive statistics on diet quality and nutrient intake of US adolescents who consume snacks, data from 2007–2018 National Health and Nutrition Examination Survey (*n* 4985)

	Daily mean	se	Nutrient requirements
HEI-2015	45·2	0·3	
MAR	59·0	0·5	
Potassium (mg/d)^*^	2252·9 mg/d	21·2	< 14 years, male: 2500
			14–18 years, male: 3000
			19 years, male: 3400
			< 19 years, female: 2300
			19 years, female: 2600
Fibre (g/d)^†^	14·7 g/d	0·2	< 14 years, male: 25·2
			14–18 years, male: 30·8
			19 years, male: 33·6
			< 14 years, female: 22·4
			14–18 years, female: 25·2
			19 years, female: 30·0
Ca (mg/d)^‡^	1026·2 mg/d	14·1	<19 years: 1300
			19 years: 1000
Vitamin D (mcg/d)^‡^	5·1 mcg/d	0·1	15
Over-consumed nutrients	143·7 %	0·8	
Added sugar (% daily energy)^§^	15·9 %	0·2	< 10 %
Saturated fat (% daily energy)^§^	11·6 %	0·08	< 10 %
Na (mg)^||^	3383·9 mg	30·0	< 14 years: 1800
			>= 14 years: 2300

HEI, Healthy Eating Index; MAR, mean adequacy ratio.

* Adequate intake (AI).

† 14 g/1000 kcal.

‡ RDA.

§ Dietary Guidelines for American (DGA).

|| Chronic Disease Risk Reduction Level (CDRR).

**Table 3:** Snack parameters as predictors of HEI-2015, MAR and over-consumed nutrients of US adolescents, data from 2007–2018 National Health and Nutrition Examination Survey

**Total snacks (*n* 4985)**						
	HEI		MAR		Over-consumed nutrients	
Snack frequency (snacks/d)	0·7	0·3^*^	0·1	0·4	3·0	0·8^**^
Snack size (kcal/snack)	−0·005	0·001^**^	−0·005	0·002^*^	0·03	0·005^**^
Snack energy density (kcal/g/snack)	−0·3	0·2	−0·7	0·3^*^	−2·3	0·6^**^
						
**Food only snacks (*n* 4757)**						
	HEI		MAR		Over-consumed nutrients	
Snack Frequency (snacks/d)	1·7	0·3^**^	1·4	0·3^**^	−2·5	0·8^**^
Snack size (kcal/snack)	−0·006	0·0009^**^	−0·006	0·002^*^	0·02	0·003^**^
Snack energy density (kcal/g/snack)	−1·1	0·2^**^	−1·5	0·3^**^	1·8	0·6^*^
						
**Beverage only snacks (*n* 3442)**						
	HEI		MAR		Over-consumed nutrients	
Snack frequency (snacks/d)	−0·1	0·6	−0·8	0·8	4·4	2·1^*^
Snack size (kcal/snack)	−0·002	0·002	−0·005	0·003	0·3	0·01^**^
Snack energy density (kcal/g/snack)	1·1	0·8	1·7	0·8^*^	−4·7	1·3^**^

HEI, Healthy Eating Index; MAR, mean adequacy ratio; mean of NAR for 4 shortfall nutrients (potassium, fibre, Ca, vitamin); over-consumed nutrients: (mean % of recommended limits for added sugar, saturated fat, Na).

Models adjusted for child age, child sex, child race and ethnicity, parental marital status, parental education status, energy misreporting and survey cycle.

* *P* < 0·05.

** *P* ≤ 0·001.

Total snack parameters were also associated with individual moderation and adequacy components of the HEI-2015 (see online supplementary material, Supplemental Table 1). Total snack frequency was positively associated with total fruits and whole fruits components, indicating greater intake, as well as refined grains, Na, and saturated fat, indicating lower intake of these moderation components. Total snack frequency was inversely associated with added sugar, but this indicates greater added sugar intake since lower scores on moderation components indicate greater intake. Total snack size was inversely associated with total fruits, whole fruits, total vegetables, greens and beans, dairy, and total protein, indicating lower intake. Total snack size was positively associated with refined grains and Na, indicating lower intake of these moderation components but inversely associated with added sugar, indicating greater intake. Finally, total snack energy density was inversely associated with total fruit, whole fruits, and dairy, indicating lower intake, and was also inversely associated with saturated fat, indicating higher intake. Total snack energy density was positively associated with whole grains, total protein, and seafood and plant protein, indicating greater intake, and added sugar, indicating lower intake. These findings were further elucidated in food only and beverage only snack models that examined associations with HEI-2015 components (see online supplementary material, Supplemental Tables 2 and 3, respectively). More frequent and lower energy-dense food only snacks were associated with higher HEI components scores on total and whole fruits, while beverage only snack size was inversely associated with most adequacy components (total fruits, whole fruits, total vegetables, greens and beans, whole grains, total protein, and seafood and plant protein) and all beverage only snacking parameters were inversely associated with added sugar indicating higher intake.

### Associations of snacking with under-consumed nutrients

Adolescents had a mean MAR score of 59·0 (0·5) indicating that average adolescent intake is just over half of dietary reference intake recommendations for the four dietary components of public health concern. Mean intakes of each of the four dietary components were below recommendations: potassium 2252·9 (21·2) mg/d, fibre 14·7 (0·2) g/d, Ca 1026·2 (14·1) mg/d and vitamin D 5·1 (0·1) mcg/d. Total snack frequency was not associated with MAR (*β* = 0·1 (0·4), *P* = 0·729), but total snack size was inversely associated with MAR (*β* = –0·005 (0·002), *P* < 0·05) as was total snack energy density (*β* = –0·7 (0·3), *P* < 0·05).

When examining snack parameters from foods only snacks, food only snack frequency was positively associated with MAR (*β* = 1·4 (0·3), *P* ≤ 0·001), while food only snack size and food only snack energy density were inversely associated with MAR (snack size *β* = –0·006 (0·002), *P* ≤ 0·001; snack energy density *β* = –1·5 (0·3), *P* ≤ 0·001). Based on beverages only, beverage only snack frequency was not associated with MAR (*β* = –0·8 (0·8), *P* = 0·354), nor was beverage only snack size (*β* = –0·005 (0·003), *P* = 0·156), but beverage only snack energy density was positively associated with MAR (*β* = 1·7 (0·8), *P* < 0·05).

### Associations of snacking with over-consumed nutrients

Mean intake of three over-consumed nutrients (added sugar, saturated fat, and Na) was 143·7 (0·8) % of recommended limits. Adolescents consumed an average of 15·9 (0·2) % of total daily calories from added sugar (158·7 (1·9)% of recommended intake), 11·6 (0·08) % of total daily calories from saturated fat (115·7 (0·8)% of recommended intake) and 3383·9 (30·0) mg of Na (156·7 (1·4)% of recommended intake). Total snack frequency and total snack size were positively associated with intakes of over-consumed nutrients (snack frequency *β* = 3·0 (0·8), *P* ≤ 0·001; snack size *β* = 0·03 (0·005), *P* ≤ 0·001), while total snack energy density was inversely associated with intake of over-consumed nutrients (*β* = –2·2 (0·6), *P* ≤ 0·001).

When examining snacks from foods only, food only snack frequency was inversely associated with intake of over-consumed nutrients (*β* = –2·5 (0·8), *P* ≤ 0·001), but food only snack size and food only snack energy density were positively associated with intake of over-consumed nutrients (snack size *β* = 0·02 (0·003), *P* ≤ 0·001; snack energy density *β* = 1·8 (0·6), *P* < 0·05). When examining beverages only, beverage only snack frequency and beverage only snack size were both positively associated with intake of over-consumed nutrients (snack frequency *β* = 4·4 (2·1), *P* < 0·05; snack size *β* = 0·03 (0·01), *P* ≤ 0·001), whereas beverage only snack energy density was inversely associated with intake of over-consumed nutrients (*β* = –4·7 (1·3), *P* ≤ 0·001).

## Discussion

Diet quality among US adolescents is notably low. Given the high prevalence of snacking and significant amount of energy contributed from snacking among US adolescents, the impact of snacking on diet quality warrants careful consideration in this age group. This research examined three key parameters of snacking: snack frequency (snacks/day), snack size (kcal/snack), and snack energy density (kcal/g/snack), and associations with three indices of diet quality – HEI-2015, intake of under-consumed nutrients of public health concern as assessed by MAR, and percentage intake of over-consumed nutrients. Results revealed that more frequent daily snack occasions were associated with higher HEI but were also associated with greater intake of over-consumed nutrients (added sugar, saturated fat, and Na). Larger snack size was associated with lower HEI, lower MAR, and greater intake of over-consumed nutrients. Total snack energy density was not associated with HEI but was inversely associated with MAR and over-consumed nutrients. These findings provide population-representative evidence that smaller, frequent, and less energy-dense snacks are associated with better diet quality among US adolescents.

To date, the literature on snacking and diet quality in adolescents remains cursory and findings from this study help to address critical gaps and methodological limitations. Work from younger paediatric populations and adults in the US suggest that snacks can help contribute to higher overall diet quality but snacking also clearly contributes to greater intake of food groups to limit including refined grains, added sugar, and fat^(15,16,39,40)^. This trend was also observed in an international sample of children and adolescents; greater snacking frequency was associated with higher micronutrient intakes, but the main sources of snack energy were desserts, sweets, and baked products^(41)^. Our work confirms these findings and further contributes important details about snacking characteristics and diet quality. First, this study contributes novel findings as it relates to snack size. Larger snack size (e.g. snacks higher in calories) was associated with lower HEI, lower MAR, and greater intake of over-consumed nutrients clearly highlighting the number of calories per snack is an important factor to consider as it relates to diet quality in adolescents. While snack size has not been robustly explored, the dietary literature more broadly has examined portion size as a key target associated with eating regulation, overall caloric intake during meals, and weight status in youth^(42,43)^. Portion sizes of snack foods available to youth have increased over recent decades alongside rates of snacking and obesity, and it is well established that larger portion sizes are associated with increased consumption^(44)^. Taken together with findings from this study, modifications to snack size such as reducing portions of energy-dense, nutrient-poor snack foods, hold promise for improving diet quality in adolescents. Related literature has also examined the context of snacking and found that factors such as snacking during screen time, consuming snacks with foods and beverages together, and peer/family influences are all associated with increased consumption (e.g. excess calories) of snack foods^(7,45)^. Learning more about the context of snacking might help inform approaches to reducing overall calories consumed at snack occasions and improve diet quality in adolescents.

Another key finding from this work highlights that snack occasions are contributing to increased intake of key public heath nutrients of concern among adolescents, namely added sugar. Adolescents who snack are consuming 158·7 (1·9) % of their recommended daily intake of added sugar and beverages consumed between meals appear to be a key contributor. However, dietary guidelines for adolescents recommend that snacks are used to promote intake of nutrient-dense foods like fruits and vegetables. Future studies should examine intervention and policy approaches that improve snack choices available to adolescents and align with profiles associated with higher diet quality. Additionally, timing of snack consumption was not explored in this study. However, adolescents tend to consume snacks late at night (e.g. after 20.00) and future work should examine timing of snack consumption that optimises diet quality in this age group^(46)^.

Snack energy density was also an important predictor of diet quality in this age group and stratified models exploring food only and beverage only snacks helped to elucidate the associations. Consistent with other studies examining energy density, findings suggest that adolescents should avoid low energy density beverages as snacks, as they do not meaningfully contribute to overall diet quality. In fact, more frequent, larger (e.g. higher calorie) and less energy-dense beverages consumed as snacks contribute to excess intake of over-consumed nutrients, most commonly in the form of added sugar. This is consistent with previous work on sugar-sweetened beverage intake in adolescents that clearly indicates sugar-sweetened beverages are the main dietary contributor of added sugar in this age group and are associated with poor overall diet quality and increased obesity risk in this age group^(47)^. However, recent work suggests that targeting sugar-sweetened beverages alone is not sufficient for improving diet quality, and more comprehensive improvements to dietary intake are needed^(48)^. Findings from the current study suggest that in addition to reducing beverages consumed between meals, increasing intake of small, frequent, low energy-dense food only snacks could contribute to higher HEI and MAR, and lower intake of over-consumed nutrients. This is consistent with previous work that has shown that low energy-dense foods like fruits and vegetables consumed as snacks can improve diet quality, while high energy-dense foods consumed as snacks contribute to poorer overall diet quality and increase caloric intake and obesity risk^(14,49)^.

This study addresses key gaps in understanding the relationship between snacking and diet quality in adolescents. Multiple parameters of snacking (snack frequency, snack size, and snack energy density) were used, and findings were conditional on adjusting for other key snacking parameters. This addresses methodological limitations noted in other studies. Additionally, analyses explored snacking contributions from foods only snacks and beverage only snacks which helped further delineate snacking contributions to overall diet quality. Multiple metrics of diet quality were employed to comprehensively examine the relationship between snacking and dietary intake. This is important as adolescents are less likely to meet dietary recommendations than any other age group and, therefore, are at greatest risk for dietary inadequacy. However, it should be noted that the Dietary Guidelines for Americans highlights additional nutrients like phosphorus, Mg, and choline as inadequate in this specific age group, which were not examined in this study, and should be considered in future work^(12)^. These data are nationally representative data and span the most recent decade, suggesting that findings are generalisable to the US adolescent population. Given that adolescents have the poorest overall diet quality among any life stage in the US, snacking is a potentially important target for shifting dietary intake to improve nutritional quality during this key phase in the life course. The findings from this study are limited by their self-reported cross-sectional design; while potential confounding variables and energy misreporting were included in the models, residual confounding and response bias are always an issue. Only participants with two recalls were included, but if 2 d of snack reporting is reflective of usual snacking patterns should also be considered when interpreting the findings. Longitudinal and experimental studies are needed to evaluate influences of snack consumption on diet quality among adolescents. Finally, this analysis adjusted for potentially important demographic correlates of snacking including gender, race and ethnicity, and household income but did not examine these relationships in detail. This is an important direction for future work and would be valuable for informing intervention approaches and dietary guidelines.

In conclusion, frequent, smaller, less energy-dense food only snacks (i.e. foods eaten between meals) should be consumed by adolescents to optimise overall diet quality, help meet MAR of under-consumed nutrients of public health concern, and reduce intake of over-consumed nutrients like added sugar, saturated fat, and Na. Frequent, larger, and less energy-dense beverages consumed as snacks are a key contributor to excess intake of over-consumed nutrients (i.e. added sugar) in adolescents and should be avoided. To inform tailored recommendations for this age group, future studies should investigate the optimal source of snack foods, the frequency and timing of snacks, and calories/portion size thresholds for this age group as well as what other factors might impact frequency and snack size (e.g. screen time, activity levels, late night consumption). These findings are among the first to provide comprehensive nationally representative data on snacking and diet quality among US adolescents.

## Supplementary material

For supplementary material accompanying this paper visit https://doi.org/10.1017/S1368980023001635. click here to view supplementary material
