# Supplementary material for: Snack frequency, size, and energy density are associated with diet quality among US adolescents
Source: Public Health Nutr. 2023 Aug 7;26(11):2374–82. doi: 10.1017/S1368980023001635 (PMC10641603; doi:10.1017/S1368980023001635)
Supplement: Supplementary file 1 [file S1368980023001635sup001.docx]

**Supplementary Figure 1**. Flow diagram of study participants.

**NHANES initial sample**

2007-2008 10,149

2009-2010 10,537

2011-2012 9,756

2013-2014 10,175

2015-2016 9,971

2017-2018 9,254

**Total 59,842**

**932** Excluded

435 Non-snackers

295 Missing HH marital status

202 Missing HH education status

**216** Excluded

79 Missing height/weight

44 Diagnosis of diabetes

93 Use of medication known to impact hunger, appetite, or weight status

**6,117** participants met inclusion criteria

**5,901** participants eligible

**4,985** final analytic sample

**53,725** Excluded

52,050 <12 years or >19 years

1,675 < 2 days of dietary recalls

**Supplementary Table 1**: Total snack parameters as predictors of HEI-2015 component subscales, data 2007-2018 National Health and Nutrition Examination Survey components (n=4,985)

| **HEI Component** |  |  | **Snack Frequency** | **Snack size** | **Snack Energy Density** |
| --- | --- | --- | --- | --- | --- |
| **Adequacy Components** | | | | | |
|  | **Max Points** | **Mean^1^ (SE)** | **Β(SE)^2^** | | |
| Total Fruits | 5 | 2.2 (0.05) | 0.2 (0.04) *** | -0.001 (0.0002)*** | -0.18 (0.03)*** |
| Whole Fruits | 5 | 2.1 (0.05) | 0.3 (0.04)*** | -0.001 (0.0002)*** | -0.2 (0.03)*** |
| Total Vegetables | 5 | 2.3 (0.03) | -0.004 (0.04) | -0.0006 (0.0001) *** | 0.03(0.03) |
| Greens and beans | 5 | 1.2 (0.04) | -0.02(0.05) | -0.0006 (0.0002)* | 0.002 (0.03) |
| Whole Grains | 10 | 2.5 (0.07) | 0.09 (0.08) | -0.0003 (0.0003) | 0.1 (0.05)* |
| Dairy | 10 | 6.6 (0.07) | -0.1 (0.07) | -0.0009 (0.0003)* | -0.1 (0.06)* |
| Total Protein | 5 | 4.0 (0.03) | -0.1 (0.03)*** | -0.0008 (0.0002)*** | 0.05 (0.02)* |
| Seafood and plant protein | 5 | 2.0 (0.05) | 0.08 (0.05) | -0.0003 (0.0002) | 0.1 (0.04)* |
| Fatty Acids | 10 | 2.0 (0.07) | -0.1 (0.1) | -0.0003 (0.0004) | 0.05 (0.07) |
| **Moderation Components** | | | | | |
| Refined Grains | 10 | 4.8 (0.07) | 0.3 (0.07)*** | 0.002 (0.0005)* | -0.1 (0.07) |
| Sodium | 10 | 4.0 (0.07) | 0.6 (0.07)*** | 0.002 (0.0004)*** | -0.006 (0.07) |
| Added Sugars | 10 | 5.8 (0.07) | -0.5 (0.07)*** | -0.003 (0.0005)*** | 0.3 (0.06)*** |
| Saturated Fat | 10 | 5.5 (0.07) | 0.2 (0.07)* | -0.0006 (0.0003) | -0.3 (0.06)*** |

*p<.05; *** p<.001; HEI= Healthy Eating Index;

^1^ Mean (SE= Standard Error) of HEI subscales

^2^ Models examining snacking parameters as predictors of HEI subscale scores; controlling for age, gender, race and ethnicity, education status, marital status, EI:EER and survey cycle

**Supplementary Table 2**: Food only snack parameters as predictors of HEI-2015 component subscales, data 2007-2018 National Health and Nutrition Examination Survey components (n=4,757)

| **HEI Component** | **Food Snack Frequency** | **Food Snack Size** | **Food Snack Energy Density** |
| --- | --- | --- | --- |
| **Adequacy Components** | | | |
|  | **Β(SE)^2^** | | |
| Total Fruits | 0.3 (0.04) *** | -0.001 (0.0001)*** | -0.38 (0.03)*** |
| Whole Fruits | 0.4 (0.04)*** | -0.0008 (0.0002)*** | -0.5 (0.03)*** |
| Total Vegetables | 0.1 (0.03)*** | -0.0006 (0.0001) *** | -0.008(0.03) |
| Greens and beans | 0.09(0.06) | -0.0003 (0.0002) | -0.02 (0.03) |
| Whole Grains | 0.2 (0.06)* | -0.0001 (0.0002) | 0.001 (0.06) |
| Dairy | 0.09 (0.06) | -0.0005 (0.0002)* | -0.1 (0.05)* |
| Total Protein | 0.05 (0.03) | -0.0007 (0.0001)*** | 0.001 (0.03) |
| Seafood and plant protein | 0.2 (0.05)*** | -0.0002 (0.0002) | 0.09 (0.04)* |
| Fatty Acids | -0.1 (0.09) | -0.00005 (0.0003) | 0.08 (0.07) |
| **Moderation Components** | | | |
| Refined Grains | 0.09 (0.08) | -0.00001 (0.0004) | 0.009 (0.07) |
| Sodium | -0.03 (0.08) | 0.001 (0.0004)* | 0.1 (0.08) |
| Added Sugars | 0.2 (0.08)* | -0.001 (0.0004)*** | -0.2 (0.06)*** |
| Saturated Fat | 0.2 (0.06)*** | -0.001 (0.0003)*** | -0.1 (0.06)* |

*p<.05; *** p<.001; HEI= Healthy Eating Index

^1^ Mean (SE= Standard Error) of HEI subscales

^2^ Models examining snacking parameters as predictors of HEI subscale scores; controlling for age, gender, race and ethnicity, education status, marital status, EI:EER and survey cycle

**Supplementary Table 3**: Beverage only snack parameters as predictors of HEI-2015 component subscales, data 2007-2018 National Health and Nutrition Examination Survey components (n=3,442)

| **HEI Component** | **Beverage Snack Frequency** | **Beverage Snack Size** | **Beverage Snack Energy Density** |
| --- | --- | --- | --- |
| **Adequacy Components** | | | |
|  | **Β(SE)^2^** | | |
| Total Fruits | 0.1 (0.1) | -0.0007 (0.0003)* | 0.1 (0.1) |
| Whole Fruits | 0.05 (0.1) | -0.0008 (0.0003)* | 0.2 (0.1) |
| Total Vegetables | 0.009 (0.06) | -0.0008 (0.0002) *** | 0.05(0.09) |
| Greens and beans | 0.01(0.08) | -0.001 (0.0002)*** | 0.1 (0.1) |
| Whole Grains | 0.05 (0.1) | -0.001 (0.0004)* | 0.2 (0.2) |
| Dairy | -0.2 (0.2) | -0.0008 (0.0007) | 0.1 (0.2) |
| Total Protein | -0.09 (0.06) | -0.0006 (0.0002)* | 0.04 (0.08) |
| Seafood and plant protein | 0.02 (0.1) | -0.0008 (0.0003)* | 0.05 (0.09) |
| Fatty Acids | 0.07 (0.2) | -0.00002 (0.0006) | -0.3 (0.2) |
| **Moderation Components** | | | |
| Refined Grains | 0.02 (0.2) | 0.004 (0.0006)*** | 0.07 (0.2) |
| Sodium | 0.4 (0.1)* | 0.003 (0.0007)*** | 0.3 (0.1) |
| Added Sugars | -0.6 (0.2)*** | -0.004 (0.0008)*** | 0.4 (0.2)* |
| Saturated Fat | 0.05 (0.2) | 0.002 (0.0005)*** | -0.3 (0.2) |

*p<.05; *** p<.001; HEI= Healthy Eating Index

^1^ Mean (SE= Standard Error) of HEI subscales

^2^ Models examining snacking parameters as predictors of HEI subscale scores; controlling for age, gender, race and ethnicity, education status, marital status, EI:EER and survey cycle
